# An innovative molecular approach towards the cost-effective entomological authentication of honey

**DOI:** 10.1038/s41538-024-00268-9

**Published:** 2024-05-02

**Authors:** Guozhi Zhang, Yanzheng Zhang, Bin Yuan, Ruth Tiang En, Shanshan Li, Huoqing Zheng, Fuliang Hu

**Affiliations:** Key laboratory of silkworm and bee resource utilization and innovation of Zhejiang Province, College of Animal Sciences, Zhejiang University, Hangzhou, 310058 China

**Keywords:** Economics, DNA

## Abstract

Honey authentication and traceability are crucial not only for economic purposes but also for ensuring safety. However, the widespread adoption of cutting-edge technologies in practical applications has been hampered by complex, time-consuming sample pre-treatment processes, the need for skilled personnel, and substantial associated expenses. This study aimed to develop a simple and cost-effective molecular technique to verify the entomological source of honey. By utilizing newly designed primers, we successfully amplified the mitochondrial 16S ribosomal RNA gene of honey bees from honey, confirming the high quality of the extracted DNA. Employing RFLP analysis with *AseI* endonuclease, species-specific restriction patterns were generated for honey derived from six closely related honey bees of the *Apis* genus. Remarkably, this method was proven equally effective in identifying heat-treated and aged honey by presenting the same RFLP profiles as raw honey. As far as we know, this is the initial research of the simultaneous differentiation of honey from closely related honey bee species using the restriction endonuclease *AseI* and mitochondrial 16S rRNA gene fragments. As a result, it holds tremendous potential as a standardized guideline for regulatory agencies to ascertain the insect origins of honey and achieve comprehensive traceability.

## Introduction

With the rapid development of the world economy, people’s requirements for food are no longer only quantity, but also increasingly stringent requirements for its safety and quality^1^. Driven by financial profit, food fraud is prevalent, and many food products have fallen into a serious crisis of confidence^2^. Therefore, in the context of the increasing global food demand, ensuring the authenticity and traceability of food has become one of the focal points of concern, and it has also become a key task in achieving the goals of global sustainable development^3^. In recent decades, honey has gained increasing popularity and the global market has been expanding steadily. However, the immense economic advantages associated with honey have unfortunately led to its emergence as the third most frequent target for adulteration, just behind olive oil and milk^4^. This poses a significant obstacle to the sustainable development of the entire industry. Therefore, ensuring the authenticity of honey has become a key focus within the industry.

Honey is a natural product of interactions between honey bees (*Apis*) and plants or honey bees and plant-sucking insects (*Hemiptera*)^5^, so a thorough characterization of its authenticity needs to be traced from both botanical and entomological sources. Most studies have focused on the plant sources of honey and have reported many methods of traceability^6–9^. In fact, the insect sources of honey are also diverse, and different honey bee species give it different values, both in terms of price and composition. However, only a small amount of research focuses on the insect sources of honey. Whilst *Apis mellifera* honey and *Apis cerana* honey dominate the global honey market in terms of volume, it is worth noting that many countries still maintain the tradition of utilizing wild honey bees to produce honey, such as the honey of giant honey bees (*Apis laboriosa* and *Apis dorsata*) accounts for 70~80% of honey production in Nepal, India, and some other Southeast Asian countries^10^. In China, the wild honey that is harvested from the forest is produced mainly by undomesticated *A. laboriosa*, *A. dorsata*, *A. florea* and *A. andreniformis*. In addition, there are significant differences in the distribution range, collection habits, and brewing methods of different honey bee species, resulting in differences in the quality of the honey produced^11–14^. The significant disparity in economic value driven by the varying nutritional value, yield, and harvesting challenges of honey from different honey bee species necessitates the importance of clarifying the entomological origin of honey. Mislabeling not only inflicts considerable damage upon the interests and rights of consumers but also undermines fair trade in the honey market. Furthermore, the economic gap of honey directly influences the strategic choices made by beekeepers when selecting honey bee species, thus affecting the overall species composition within beekeeping^15^. Therefore, the identification of the insect source of honey is essential for the stable and orderly development of the honey industry. Unfortunately, based on the appearance and flavor of honey and the current honey standard^5,16^, we hardly distinguish the honey of different honey bee species. Hence, establishing an expeditious, reliable, and cost-efficient technique for ascertaining the entomological provenance of honey is eagerly awaited in current market production.

In recent years, researchers have also been attempting to utilize various traceability techniques for declaring the insect origins of honey^17,18^. It should be noted that among these methods, DNA-based approaches have demonstrated remarkable stability, wide applicability, and extraordinary specificity in identifying the entomological origins of honey. During the process of honey bees collecting and processing nectar to form honey, the presence of fragmented honey bee cells and tissues provides a precise perspective for utilizing honey bee DNA in the identification of the insect source of honey. Recent studies have demonstrated the successful use of species-specific primers designed based on several target genes: the tRNA^leu^-cox2 gene in *A. cerana*^19^, the Major Royal Jelly Protein 2 (MRJP2) gene in both *A. cerana* and *A. mellifera*^20^, and the NADH dehydrogenase 2 (ND2) gene in *A. cerana*, *A. mellifera*, and *A. dorsata*^21^. Nevertheless, challenges persist in this type of analysis. The range of identifiable species within a single Polymerase Chain Reaction (PCR) reaction is limited, and designing species-specific primers for multiple closely related species poses notable difficulties. Real-time PCR coupled with High-Resolution Melting (HRM) analysis, which has been used to differentiate *A. cerana* honey from *A. mellifera* honey^19^, as well as to identify honey from various *A. mellifera* subspecies^22,23^, is a noteworthy development. Despite the promising results, this method is not exempt from limitations, including the necessity of costly equipment and the establishment of a melting curve database to serve as a standard reference for unknown samples^24^. The Polymerase Chain Reaction-Restriction Fragment Length Polymorphism (PCR-RFLP) technique, as a classical PCR technique, has been widely used in food authenticity identification due to its low cost, high accuracy, easy testability (especially suitable for situations where complex and expensive equipment is not available other than simple and inexpensive PCR amplifiers and gel electrophoresis equipment), intuitive results (complex calculations and analyses free)^25–27^. From this point of view, this technique overcomes the limitations of other molecular methods and has obvious superiority in the promotion of practical applications. The above-commented characteristics make PCR-RFLP the method of choice for differentiating between closely related species. Regrettably, it has not yet been applied to the identification of the entomological origin of honey.

To our knowledge, there is currently no simple and feasible method to differentiate honey from the six species of honey bees in the genus *Apis*. In this study, we explore the feasibility of using the stable and cost-effective mainstream food traceability technique PCR-RFLP to identify the insect source of honey from various biological origins. To enhance the accessibility and applicability of the method, we utilized specimens of six honey bee species (*A. mellifera*, *A. cerana*, *A. laboriosa*, *A. dorsata*, *A. florea*, and *A. andreniformis*) from diverse geographic origins in China, to develop the identification technique. Subsequently, the developed methodology was expanded to the analysis of genuine and processed honeys. This method only requires a pair of primers and a restriction endonuclease to simultaneously identify the different honey bee species origins of honey. This significant endeavor provides a reference for the traceability of food products with complex biological origins.

## Results and discussion

### Quality of DNA extracts and PCR products

Honey DNA is a mixture of DNA from many different organisms, such as honey bees, plants, and microorganisms^28,29^ and it can be used to authenticate the plant species that the honey bees foraged from^30,31^, distinguish the geographical origin of honey^32,33^, trace the entomological origin of honey^17,34^, and monitor the health of the honey bee colony^35–37^. The quality of DNA plays a significant role in verifying the authenticity of food products using molecular methods, and it can be influenced by several factors, such as the manufacturing process, extraction technique, and composition of the food matrix, among various other considerations^25^. In this study, we extracted DNA from honey bee tissues and honey and determined their quality. The DNA extracted from all honey bee tissues had a concentration of 19.5~556.6 ng/μL and a purity of 1.84~2.19, while honey DNA had a concentration of 8.5~1585.6 ng/μL and a purity of 1.76~2.25. This result suggests that despite the challenges posed by high sugar content and plant secondary metabolites in honey, which can hinder honey DNA extraction and PCR amplification^7,38^, the commercially available silica-column extraction-based DNA extraction kits employed in this study still exhibit the potential to extract high-quality DNA from honey. This is supported by the fact that the DNA quality obtained in this study surpassed that of previous studies^19,23^.

**Fig. 1:**
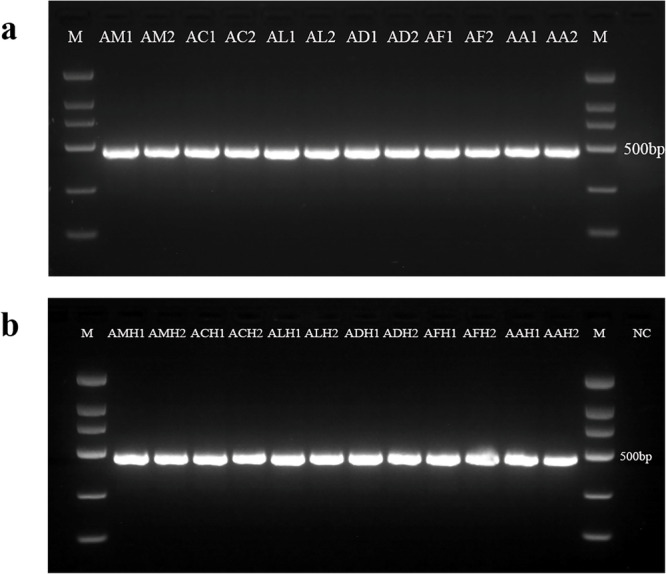
Agarose gel electrophoresis of PCR products of honey bee species (or honey) obtained with 16S rRNA-F/16S rRNA-R primers. **a** PCR products from honey bee species. Lane M: 100 bp marker. AM1, AM2: *A. mellifera*; AC1, AC2: *A. cerana*; AL1, AL2: *A. laboriosa*; AD1, AD2: *A. dorsata*; AF1, AF2: *A. florea*; AA1 and AA2: *A. andreniformis*; **b** PCR products from honey. Lane M: 100 bp marker. AMH1, AMH2: *A. mellifera* honey; ACH1, ACH2: *A. cerana* honey; ALH1, ALH2: *A. laboriosa* honey; ADH1, ADH2: *A. dorsata* honey; AFH1, AFH2: *A. florea* honey; AAH1 and AAH2: *A. andreniformis* honey; NC negative control.

Considering that the complex substances in honey may degrade and fragment the DNA remaining in honey^39^, and that shorter fragments contain insufficient genetic information, we finally designed a pair of primers (16S rRNA-F/16S rRNA-R) for amplifying a 16S rRNA gene fragment of 470~479 base pair (bp). The expected PCR products were amplified successfully in six *Apis* honey bee species (Fig. 1a) and their honey samples (Fig. 1b) using the above primers. Notably, the positive PCR results indicate that the designed primers have better specificity and honey DNA extracts do not contain PCR reaction inhibitors as well as include entomological DNA of sufficient quality.

### Data analysis of 16S rRNA gene sequences

**Fig. 2:**
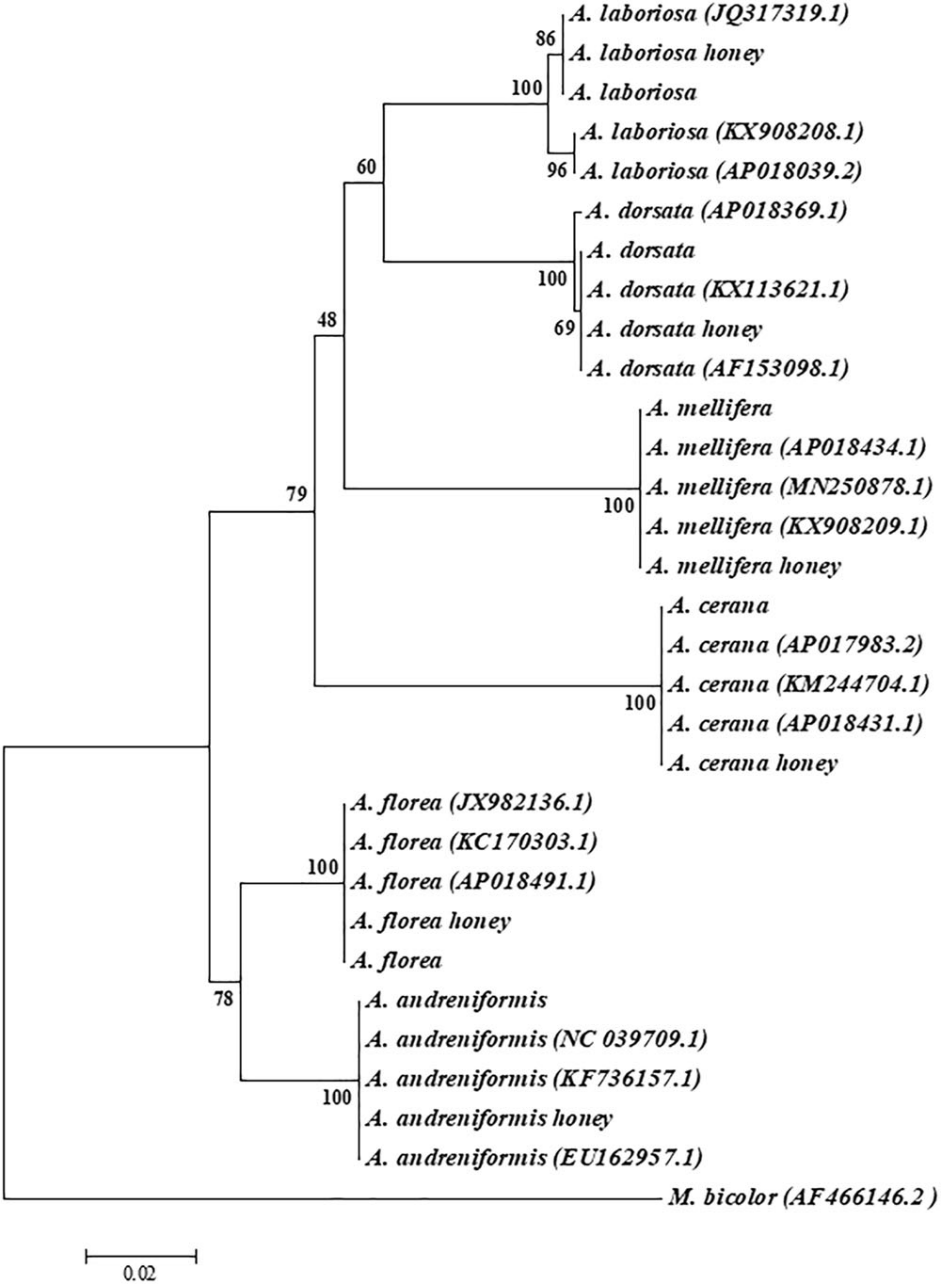
A phylogenetic tree was constructed based on the alignment of 470~479 bp mitochondrial 16S rRNA gene sequences obtained from honey bees and honeys. The construction was performed using the neighbor-joining method with the Kimura 2-parameter model. The sequences with NCBI accession numbers represent published honey bee sequences retrieved from GenBank. The numbers displayed above the branches indicate the percentage bootstrap values generated from 1000 replicates.

Owing to the high abundance and copy number of mitochondrial DNA (mtDNA) present in total cellular DNA, the amplification of PCR is considerably more effective in comparison to those of nuclear DNA^40^. Inherited through the maternal lineage and not subject to recombination^41^, mtDNA is widely regarded as an invaluable resource for phylogenetic investigations. The 16 S rRNA gene stands as the extensively employed mtDNA gene in preceding phylogenetic investigations of honey bees, closely followed by the cytochrome c oxidase subunit I (COI) gene. Phylogenetic analysis based on honey bee mitochondrial 16 S rRNA and COI gene sequences has been successfully used to identify honey entomological origin^42^. In this study, 470~479 bp of the mitochondrial 16 S rRNA gene was successfully amplified in all the six honey bee species and their honey, and also the expected sequence of those PCR products was confirmed by sequencing. We aligned these obtained sequences with the sequences published in the National Centre for Biotechnology Information (NCBI) GenBank by using the BLAST to identify its species. Sequences were exactly matched with the name of respective honey bee species as collected and were published at NCBI GenBank with accession number PP621844 (*A. mellifera*), PP621799 (*A. cerana*), PP621846 (*A. laboriosa*), PP621845 (*A. dorsata*), PP621848 (*A. florea*) and PP621849 (*A. andreniformis*).The results showed that all the sequences in this study matched to previous sequences of the same species. The sequences of *A. mellifera* and *A. mellifera* honey show a 100% similarity with *A. mellifera* (AP018434.1, MN250878.1, and KX908209.1). The sequence similarity search of *A. cerana* and *A. cerana* honey sequences shows 100% similarity to that of *A. cerana* (AP017983.2, KM244704.1, and KF572604.1). Similarly, the sequences of *A. laboriosa* and *A. laboriosa* honey show over 99% similar with *A. laboriosa* (JQ317319.1, KX908208.1, and AP018039.2). The sequences derived from both *A. dorsata* and *A. dorsata* honey exhibit an absolute resemblance of 100% to *A. dorsata* (KX113621.1, AF153098.1, and NC_037709.1), the sequences of *A. florea* and *A. florea* honey present 100% identity with *A. florea* (AP018491.1, JX982136.1, and KC170303.1) and the *A. andreniformis* sequence and *A. andreniformis* honey sequence display a similarity of over 99% with that of *A. andreniformis* (EU162957.1, KF736157.1, and NC_039709.1). The pairwise genetic distance between the six honey bee species in genus *Apis* was calculated by Kimura-2-parameter model, based on the sequences generated from the current study. The result shows that all the species were genetically different between and among themselves with a range of 3.5%~12.1% nucleotide differences for the mitochondrial 16S rRNA gene. Using the mitochondrial 16S rRNA gene sequences of honey bees and honey samples in this study and the same species in NCBI to build a phylogenetic tree (Fig. 2), the results show that samples belonging to the same honey bee species have fallen into the same clade and are separated from other species. Therefore, the honey from the six honey bee species in the genus *Apis* can be distinguished by the mitochondrial 16S rRNA gene sequence analysis.

### PCR-RFLP

**Fig. 3:**
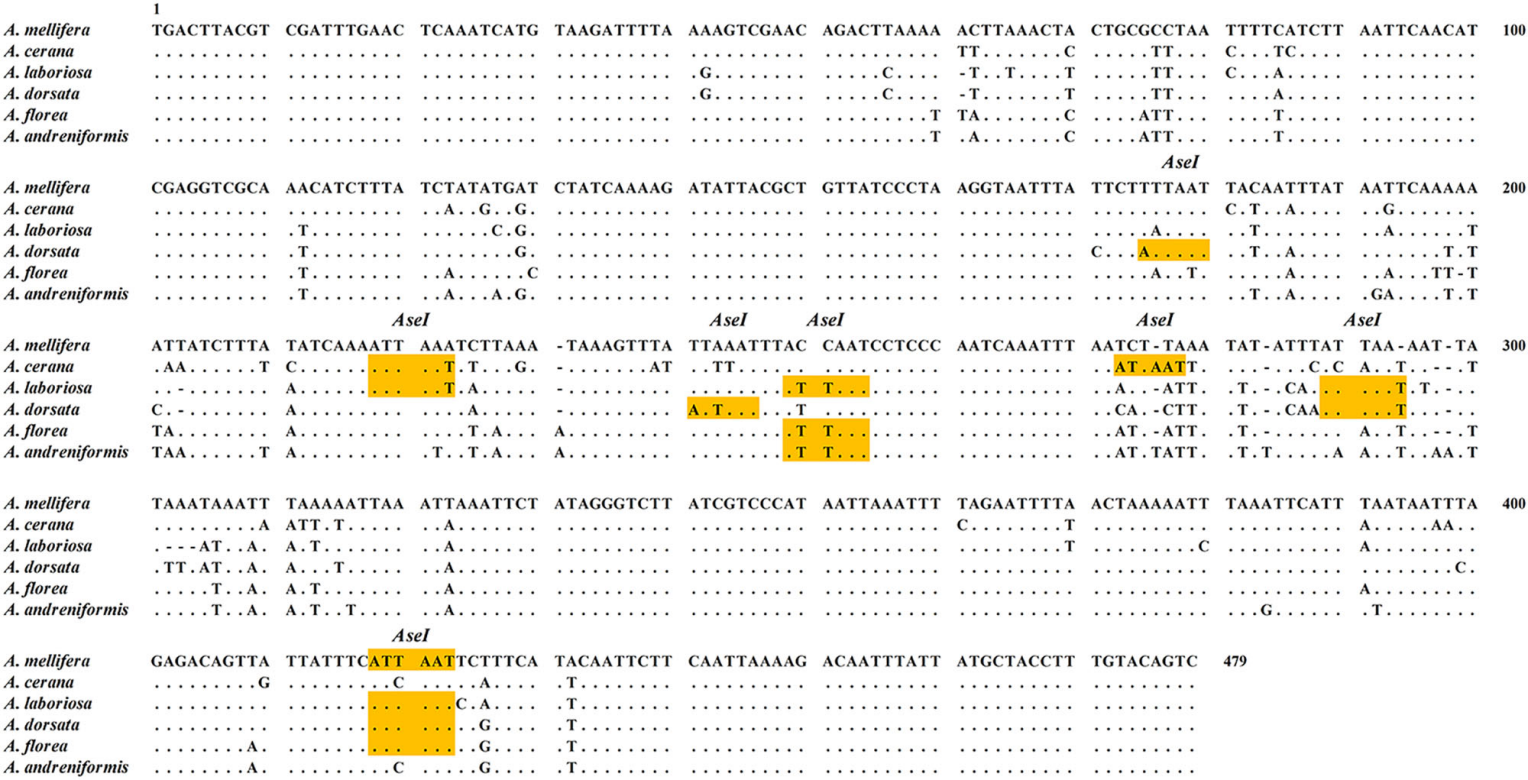
Aligned DNA sequences of the mitochondrial 16S rRNA gene obtained from six honey bee species during the present study. The restriction site for *AseI* is highlighted.

**Fig. 4:**
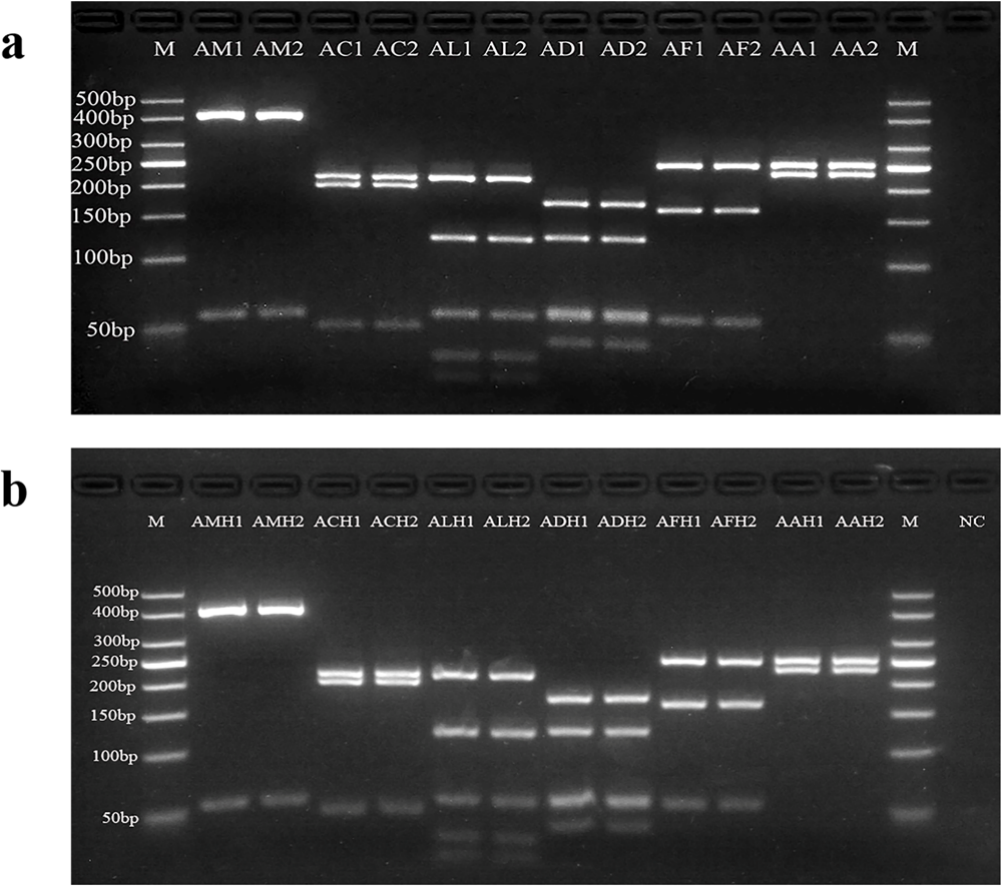
RFLP patterns of PCR products amplified from honey bee (or honey) using *AseI.* **a** PCR-RFLP profile of honey bee. Lane M: 50 bp marker. AM1, AM2: *A. mellifera*; AC1, AC2: *A. cerana*; AL1, AL2: *A. laboriosa*; AD1, AD2: *A. dorsata*; AF1, AF2: *A. florea*; AA1 and AA2: *A. andreniformis*. **b** PCR-RFLP profile of honey. Lane M: 50 bp marker. AMH1, AMH2: *A. mellifera* honey; ACH1, ACH2: *A. cerana* honey; ALH1, ALH2: *A. laboriosa* honey; ADH1, ADH2: *A. dorsata* honey; AFH1, AFH2: *A. florea* honey; AAH1 and AAH2: *A. andreniformis* honey; NC: negative control.

Striving to minimize the count of restriction endonucleases, while ensuring the successful attainment of identification objectives, is the foundational aspect that enhances the advantages of the PCR-RFLP method. Therefore, the selection of target gene size and restriction enzymes is crucial. Wilwet et al.^43^ successfully applied PCR-RFLP assay for identification of four commercially important shrimp species by amplification of 530 bp DNA fragment from 16S rRNA gene, followed by digestion with *Tsp5091* restriction enzyme. After conducting preliminary analysis and exploration, we ultimately opted for the utilization of the 16S rRNA gene as the target gene in present study. In order to select suitable commercial restriction enzymes, the obtained nucleotide sequences were assessed using NEBcutter V2.0. The sequences with recognition sites of *AseI* are presented in Fig. 3 and the amplified PCR product was then cleaved using the restriction enzyme, *AseI*. Figure 4 shows PCR-RFLP pattern of amplified PCR products cleaved by *AseI* from honey bee tissue and honey. A single restriction enzyme, *AseI*, was capable of distinguishing all six honey bee species and their honey samples by presenting different DNA fragment sizes. The restriction enzyme cleaved the DNA products of *A. mellifera* and *A. mellifera* honey into 2 major fragments of 414 bp and 60 bp sizes. On the other hand, amplicon of *A. cerana* and *A. cerana* honey was fragmented into 3 major fragments of 219 bp, 202 bp and 54 bp sizes. The amplified products of *A. laboriosa* and *A. laboriosa* honey were cleaved to form 3 major bands of 217 bp, 125 bp, and 60 bp sizes. The enzyme, *AseI*, digested the amplicon of *A. dorsata* and *A. dorsata* honey into three major bands of 175 bp, 128 bp, and 60 bp sizes. The amplified products of *A. florea* and *A. florea* honey were cut into 3 main fragments of 249 bp, 165 bp, and 60 bp sizes by restriction enzyme. In addition, the PCR products amplified from *A. andreniformis* and *A. andreniformis* honey were fragmented by restriction digestion into two major bands of 250 bp and 229 bp sizes (Table 1). The obtained results clearly indicated that the restriction enzyme *AseI* can generate species-specific RFLP patterns and then establish the entomological sources of honey.

**Table 1:** Expected and obtained sizes of cleaved DNA fragments of honey from six honey bee species in genus *Apis* digested with a single restriction endonuclease, *AseI*

Entomological	Seq. size (bp)	*AseI*	
origin of honey		Expected	Obtained
*A. mellifera*	474	414, 60	414, 60
*A. cerana*	475	219, 202, 54	219, 202, 54
*A. laboriosa*	470	217, 125, 60, 38, 30	217, 125, 60, <50
*A. dorsata*	473	175, 128, 64, 60, 46	175, 128, 60, <50
*A. florea*	474	249, 165, 60	249, 165, 60
*A. andreniformis*	479	250, 229	250, 229

### Applicability of the method developed in this study

**Fig. 5:**
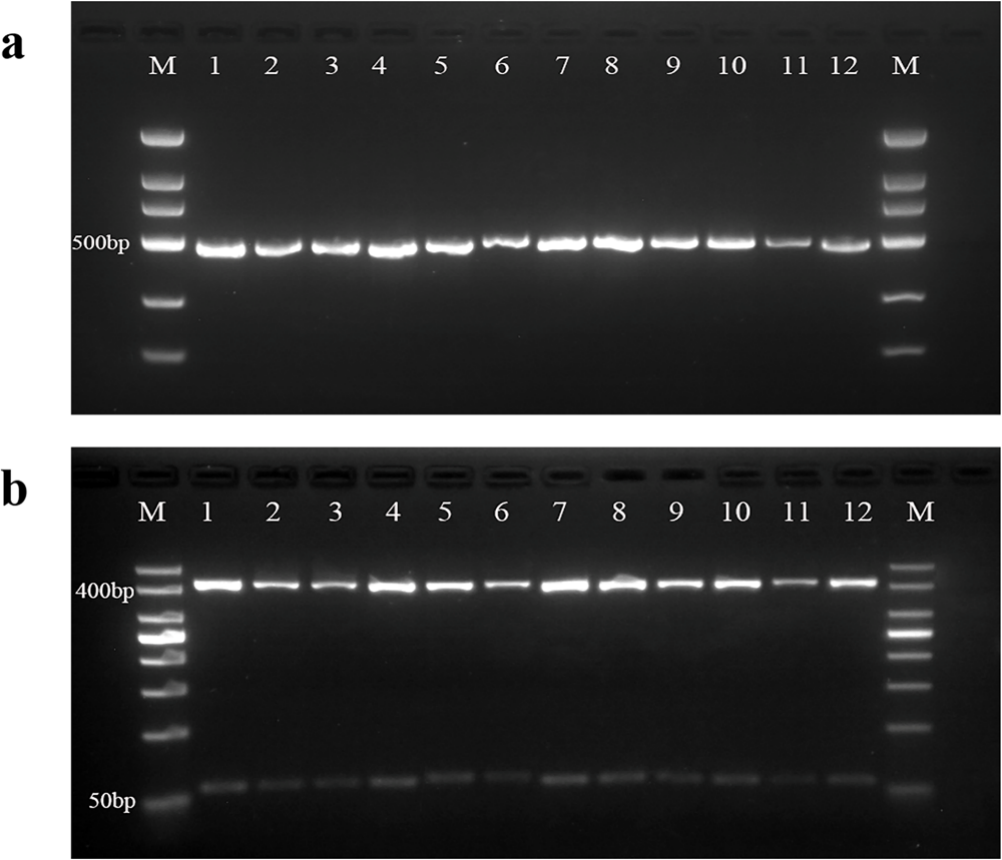
Analysis of PCR products (or PCR-RFLP profile) obtained from *A. mellifera* honey under different processing conditions. **a** PCR products of *A. mellifera* honey. Lane M: 100 bp marker. 1: raw; 2: room temperature for 1 year; 3. room temperature for 5 years; 4: −20 °C for 6 years; 5: −20 °C for 8 years; 6: crystalline honey; 7: 60 °C for 1 h; 8: 70 °C for 1 h; 9: 80 °C for 1 h; 10: 90 °C for 1 h; 11: 100 °C for 1 h; 12: commercial honey. **b** PCR-RFLP profile of *A. mellifera* honey. Lane M: 50 bp marker. 1: raw; 2: room temperature for 1 year; 3. room temperature for 5 years; 4: −20 °C for 6 years; 5: −20 °C for 8 years; 6: crystalline honey; 7: 60 °C for 1 h; 8: 70 °C for 1 h; 9: 80 °C for 1 h; 10: 90 °C for 1 h; 11: 100 °C for 1 h; 12: commercial honey.

To further evaluate the applicability of the PCR-RFLP protocol developed in this study, we conducted research on *A. mellifera* honey in different states (heated, long-term stored, crystallized, and commercial). This was done to address potential issues such as thermal processing, long-term storage, and crystallization, which may affect the entomological traceability of honey. Agarose gel electrophoresis results (Fig. 5) demonstrated the effectiveness of the method even after honey had been stored at room temperature for 5 years, chilled at −20 °C for 8 years, heated at 100 °C for 1 hour, or undergone crystallization, as well as when analyzing commercial honey. In all tested honey states, the PCR-RFLP profiles showed a similarity comparable to that of raw honey, using the same restriction enzyme, *AseI*. Additionally, the process of cooking or autoclaving had no impact on the generation of PCR-RFLP profiles or analytical accuracy^40^. These findings are in line with previous studies, such as those by Wang et al.^44^ and Zeng et al.^45^ which have established that nucleic acid-based analyses are less susceptible to disruption or interference caused by various food processing techniques.

By analyzing the insect origin of honey, it helps to establish a link between the entomological profiles of honey samples and specific plant species or floral origins. Through entomological certification, it becomes possible to verify and validate claims regarding the botanical origin of honey. Moreover, the geographic origin of honey can also be assessed through entomological analysis^22,29^. Different regions have distinct honey bee species, influenced by local environmental factors, beekeeping practices, and nature reserves for honey bee species. Researchers can identify specific honey bee species that are prevalent in certain geographical areas, allowing for the differentiation of honey based on its entomological characteristics. This provides valuable information for geographical certification, enabling consumers to identify the specific regions from which the honey originates. The high price of wild honey compels people to proactively pay attention to the conservation of wild bee species, ensuring the sustainable acquisition of their honey. This is also crucial for plant pollination, as well as for maintaining ecological balance and biodiversity. In summary, entomological certification of honey not only protects the rights of consumers and producers, but also serves as an essential tool in corroborating the botanical and geographic origins of honey. Further, it is also of great benefit to the conservation and development of honey bee resources and to the ecological value of honey bee pollination of plants.

To date, there are no standard guidelines for certifying the insect origin of honey. Moreover, there is increasing interest in applying genetic tools to the study of traceability of the food chain^46^. Within this investigation, a cost-effective, distinctive, and single enzyme PCR-RFLP reference profile for the precise identification of the entomological sources of honey from six honey bee species belonging to the genus *Apis*, was developed based on mitochondrial 16S rRNA gene fragment. By employing the PCR-RFLP protocol outlined in this study, the entomological origin of honey can be reliably identified, regardless of its state or potential processing treatments. The focus of this method is on accurately identifying the entomological origin of unknown honey using just one pair of primers and a single restriction endonuclease. Furthermore, it empowers us to assess the current labeling practices associated with these products in the market and might prove to be a useful tool for honey traceability. The method has successfully enabled the identification of honey from levels of several closely related honey bee species, and similarly, it provides a reference for the identification of honey from different honey bee subspecies and even breed sources.

## Methods

### Collection of honey bees and honey samples

Specimens of honey bee workers (as the positive control) from different species of *Apis* were used in present work: *A. mellifera* from Hangzhou (*n* = 5), Jinhua (*n* = 1), Shaoxing (*n* = 2) and Beijing (*n* = 2); *A. cerana* from Hangzhou (*n* = 5), Jiaxing (*n* = 2), Yichun (*n* = 2) and Nanchang (n = 2); *A. laboriosa* from Honghe Hani and Yi Autonomous Prefecture (*n* = 5), Lincang (*n* = 3) and Dehong Dai and Jingpo Autonomous Prefecture (*n* = 5); *A. dorsata* from Dai Autonomous Prefecture of Xishuangbanna (*n* = 10); *A. florea* from Dai Autonomous Prefecture of Xishuangbanna (*n* = 6) and Chongzuo (*n* = 2); *A. andreniformis* from Dai Autonomous Prefecture of Xishuangbanna (*n* = 3). Twenty honey bees were randomly selected from a single colony and submerged in a 50 mL centrifuge tube containing anhydrous ethanol, thus forming one honey bee sample. All honey bee samples were transferred with ice bags and then stored at −20 °C until analysis.

In total, 148 authentic honey samples with known entomological origin were collected from the species of *A. mellifera* (*n* = 30), *A. cerana* (*n* = 30), *A. laboriosa* (*n* = 30), *A. dorsata* (*n* = 30), *A. florea* (*n* = 26) and *A. andreniformis* (*n* = 2) by beekeepers during the period 2014 ~ 2022 in China. The detailed source information for honey can be found in Supplementary Table 1-6. Due to the small colony size, low honey production and limited distribution of *A. andreniformis*, coupled with the rainy climate, we only obtained two samples of *A. andreniformis* honey. Most honey samples were preserved at 4 °C prior to analysis. A few honey samples were stored at room temperature for validation of the developed method. High-fructose corn syrup was utilized as a negative control to represent non-authentic honey.

### DNA extraction

Pre-treatment was required before extracting DNA from both honey bee samples and honey samples. The thorax of 1 or 2 honey bees after drying with a paper towel was homogenised in 150 μL of PBS buffer with five zirconia beads (3 mm bead size) using Tissuelyser-24 (Shanghai Jingxin Industrial Development Co., Ltd., Shanghai, China) for 1 min at 200 Hz. The suspension was centrifuged at 10,000 rpm for 1 min, the supernatant was removed carefully and the pellet was stored at −20 °C until DNA extraction.

A pre-treatment of honey samples was performed according to a previous study^19^ with slight modifications. 15 g of each honey sample was diluted in 30 mL of distilled water and heated to 45 °C for 10 min. After homogenization and centrifugation at 10,000 rpm for 30 min, the supernatant was discarded, and the pellet was resuspended in 1 mL of distilled water. The mixture was centrifuged again (12,000 rpm for 15 min), the supernatant was discarded and the pellet was stored at −80 °C until the DNA extraction was performed.

DNA from both honey bees and honey pellets was extracted using TIANamp Genomic DNA Kit (Tiangen Biochemical Technology Co., Ltd., Beijing, China), according to the manufacturer’s instructions. The extracted DNA was detected by a NanoDrop 2000 spectrophotometer (Thermo Fisher Scientific, USA) for DNA purity (A260/A280 nm) and concentration.

### Primer design

The mitochondrial 16 S rRNA gene sequences of *A. mellifera* (NC_051932.1), *A. cerana* (NC_014295.1), *A. laboriosa* (NC_036155.2), *A. dorsata* (NC_037709.1)*, A. florea* (NC_021401.1) and *A. andreniformis* (NC_039709.1) were used to design the universal primers. The sequences of these honey bee species were retrieved from GenBank and aligned using MEGA7.0 software (https://www.megasoftware.net/). The conserved regions in this gene were used to design universal primers by Primer Premier 5 software (Premier Biosoft, Palo Alto, CA, USA). The designed primers were synthesised by Tsingke Biotech (Beijing, China).

### Amplification of the 16S rRNA gene fragment

PCR was carried out to amplify the 470 ~ 479 bp of the mitochondrial 16 S rRNA gene using the newly designed universal primers 16 S rRNA-F (5′-TGACTTACGTCGATTTGAAC-3′) and 16 S rRNA-R (5′-GACTGTACAAAGGTAGCATAAT-3′) using Model MG96+ Peltier Thermal Cycler (LongGene Scientific Instruments Co., Ltd., Hangzhou, China). The PCR reaction mixture contained 2 μL DNA template, 10 μL 2 × Taq PCR StarMix (GenStar, Beijing, China), 0.5 μL each primer (10 μM), and 7 μL molecular grade water. The amplification conditions were as follows: initial denaturation at 94 °C for 2 min, followed by 30 cycles of amplification (94 °C for 30 s, 50 °C for 30 s, and 72 °C for 30 s), and a final extension at 72 °C for 5 min. The amplified products (5 μL) were analyzed by gel electrophoresis on 2% agarose gel containing TS-GelRed 1X (Tsingke Biotechnology Co., Ltd., Beijing, China) and visualized using JS-680D gel documentation system (Peiqing, Shanghai, China). A DNA marker (Sangon Biotech, Shanghai, China) with a molecular weight range of 100 ~ 2000 bp was used as a size comparison.

### Sequencing, data analysis, and selection of the restriction enzyme

The PCR products were submitted to Tsingke Biotech for Sanger sequencing, and sequences were identified by BLAST (https://www.ncbi.nlm.nih.gov/BLAST). The sequences obtained from the present study were published in NCBI and published with GenBank accession numbers: PP621844, PP621799, PP621846, PP621845, PP621848, and PP621849. The nucleotide sequences were aligned and edited using MEGA7.0. Based on partial 16 S rRNA gene sequences of six honey bee species, the neighbor-joining (NJ) phylogenetic tree was created using Kimura 2-parameter model 1000 bootstrap replicates, and the pairwise genetic distance was calculated using MEGA7.0 software^45^. The NEBcutter version 2.0 online software (http://nc2.neb.com/NEBcutter2/) was used to select a suitable restriction enzyme^47^ that can generate the respective restriction DNA fragments for each honey bee species with band sizes and numbers easily distinguishable on agarose gels based on the 470~479 bp mitochondrial 16 S rRNA sequences.

### PCR-RFLP analysis

*AseI* restriction enzyme was used to digest the PCR products above. This enzyme was obtained from an *Escherichia coli* strain that carries the *AseI* (New England Biolabs, Ipswich, MA, USA) gene of *Aquaspirillum serpens* (ATCC 12638) and it recognizes AT^TAAT sites and cuts optimally at 37 °C in NEBuffer r3.1. For PCR-RFLP assays, the digestion reaction volume was 25 μL consisting of 10 μL of amplified PCR product, 2.8 μL NEBuffer r3.1 (10 X), 1 μL (10U) of *AseI* restriction endonuclease, and 11.2 μL of molecular grade water. The mixtures were homogenized and incubated at 37 °C for 2 h. In addition to the agarose gel concentration of 4%, cleaved PCR products (14 μL) were electrophoresed and visualized by a gel documentation system. The length of cleaved PCR products was determined by a 50 bp DNA ladder (Tsingke Biotechnology Co., Ltd., Beijing, China). In this study, all gels derive from the same experiment, and they were processed in parallel.

### Suitability test

Given that *A. mellifera* honey is widely produced globally and often used to counterfeit and adulterate honey from other honey bee species, we selected it as our primary test subject. Therefore, the suitability test of the method developed in this study was carried out using *A. mellifera* honey samples with different storage times and temperature (1 year and 5 years at room temperature, 6 years and 8 years at −20 °C), thermal processing (60 °C, 70 °C, 80 °C, 90 °C and 100 °C water-bath heating for 1 h) and other factors (crystallized and commercial honey) for PCR-RFLP analysis as described above. There were three honey samples for each condition mentioned above. The commercial honey samples were purchased from Zhejiang Fucide Biotechnology Co., Ltd (Zhejiang, China).

### Reporting summary

Further information on research design is available in the Nature Research Reporting Summary linked to this article.

### Supplementary information

Supplementary Information

Reporting summary

## Data Availability

The authors declare that all data supporting the findings of this study are available in the paper and supplementary information.

## References

[1] He Y (2021). Detection of adulteration in food based on nondestructive analysis techniques: a review. Crit. Rev. Food Sci. Nutr..

[2] Esteki M, Regueiro J, Simal-gándara J (2019). Tackling fraudsters with global strategies to expose fraud in the food chain. Compr. Rev. Food Sci. Food Saf..

[3] Lillford P, Hermansson A-M (2021). Global missions and the critical needs of food science and technology. Trends Food Sci. Tech..

[4] Moore JC, Spink J, Lipp M (2012). Development and application of a database of food ingredient fraud and economically motivated adulteration from 1980 to 2010. J. Food Sci..

[5] Codex Alimentarius Commission. Revised codex standards for honey. *Codex Standard* 12–1981, Rev.2 (2001).

[6] Biswas A, Naresh K, Jaygadkar SS, Chaudhari SR (2023). Enabling honey quality and authenticity with NMR and LC-IRMS based platform. Food Chem..

[7] Soares S, Amaral JS, Oliveira MBPP, Mafra I (2017). A comprehensive review on the main honey authentication issues: production and origin. Compr. Rev. Food Sci. Food Saf..

[8] Tsagkaris AS (2021). Honey authenticity: analytical techniques, state of the art and challenges. RSC Adv..

[9] Yu W (2023). Identification of the botanical origins of honey based on nanoliter electrospray ionization mass spectrometry. Food Chem..

[10] Woyke J (2008). Comparison of defense body movements of *Apis laboriosa*, *Apis dorsata dorsata* and *Apis dorsata breviligula* honey bees. J. Insect Behav..

[11] Kek SP, Chin NL, Yusof YA, Tan SW, Chua LS (2018). Classification of entomological origin of honey based on its physicochemical and antioxidant properties. Int. J. Food Prop..

[12] Wu J (2022). Bioactive characterization of multifloral honeys from *Apis cerana cerana*, *Apis dorsata*, and *Lepidotrigona flavibasis*. Food Res. Int..

[13] Yang W (2021). The botanical sources, entomological proteome and antibiotic properties of wild honey. Innov. Food Sci. Emerg. Technol..

[14] Yap SK, Chin NL, Shah NNAK, Than LTL, Niranjan K (2022). Physicochemical characteristics, antioxidant properties and bacterial profiling of three Malaysian honey varieties: a study using multivariate analysis. J. Sci. Food Agric..

[15] Lee H, Sumner DA, Champetier A (2019). Pollination markets and the coupled futures of almonds and honey bees: simulating impacts of shifts in demands and costs. Am. J. Agr. Econ..

[16] European Commission (2002). Council Directive 2001/110/EC of 20 December 2001 relating to honey. J. Eur. Commun..

[17] Mohamadzade Namin S, Ghosh S, Jung C (2023). Honey quality control: review of methodologies for determining entomological origin. Molecules.

[18] Wang X (2022). Systematic review of the characteristic markers in honey of various botanical, geographic, and entomological origins. ACS Food Sci. Technol..

[19] Soares S (2018). Novel diagnostic tools for Asian (*Apis cerana*) and European (*Apis mellifera*) honey authentication. Food Res. Int..

[20] Zhang Y-Z (2019). Authentication of *Apis cerana* honey and *Apis mellifera* honey based on major royal jelly protein 2 gene. Molecules.

[21] Mohamadzade Namin S, Yeasmin F, Choi HW, Jung C (2022). DNA-based method for traceability and authentication of *Apis cerana* and *A. dorsata* honey (Hymenoptera: Apidae), using the NADH dehydrogenase 2 gene. Foods.

[22] Honrado M, Lopes AR, Pinto MA, Amaral JS (2022). A novel real-time PCR coupled with high resolution melting analysis as a simple and fast tool for the entomological authentication of honey by targeting *Apis mellifera* mitochondrial DNA. Food Res. Int..

[23] Soares S (2019). Towards honey authentication: differentiation of *Apis mellifera* subspecies in European honeys based on mitochondrial DNA markers. Food Chem..

[24] Madesis P, Ganopoulos I, Sakaridis I, Argiriou A, Tsaftaris A (2014). Advances of DNA-based methods for tracing the botanical origin of food products. Food Res. Int..

[25] Baptista M, Cunha JT, Domingues L (2021). DNA-based approaches for dairy products authentication: a review and perspectives. Trends Food Sci. Tech..

[26] Cermakova E (2023). Identification of fish species and targeted genetic modifications based on DNA analysis: state of the art. Foods.

[27] Vesterlund S-R, Sorvari J, Vasemagi A (2014). Molecular identification of cryptic bumblebee species from degraded samples using PCR-RFLP approach. Mol. Ecol. Resour..

[28] Galanis A (2022). Bee foraging preferences, microbiota and pathogens revealed by direct shotgun metagenomics of honey. Mol. Ecol. Resour..

[29] Pathiraja D, Cho J, Kim J, Choi I-G (2023). Metabarcoding of eDNA for tracking the floral and geographical origins of bee honey. Food Res. Int..

[30] Laube I (2010). Development of primer and probe sets for the detection of plant species in honey. Food Chem..

[31] Özkök A (2023). Comparing the melissopalynological and next generation sequencing (NGS) methods for the determining of botanical origin of honey. Food Control.

[32] Khansaritoreh E (2020). Employing DNA metabarcoding to determine the geographical origin of honey. Heliyon.

[33] Liu S (2022). Tracing the origin of honey products based on metagenomics and machine learning. Food Chem..

[34] Moškrič A, Mole K, Prešern J (2021). EPIC markers of the genus *Apis* as diagnostic tools for detection of honey fraud. Food Control.

[35] Bovo S, Utzeri VJ, Ribani A, Cabbri R, Fontanesi L (2020). Shotgun sequencing of honey DNA can describe honey bee derived environmental signatures and the honey bee hologenome complexity. Sci. Rep..

[36] Revainera P (2020). Molecular detection of bee pathogens in honey. J. Insects Food Feed.

[37] Salkova D (2022). Molecular detection of *Nosema* spp. In honey in Bulgaria. Vet. Sci..

[38] Soares S, Amaral JS, Oliveira MBPP, Mafra I (2015). Improving DNA isolation from honey for the botanical origin identification. Food Control.

[39] Utzeri VJ, Ribani A, Fontanesi L (2018). Authentication of honey based on a DNA method to differentiate *Apis mellifera* subspecies: application to Sicilian honey bee (*A. m. siciliana*) and Iberian honey bee (*A. m. iberiensis*) honeys. Food Control.

[40] Sun Y-L, Lin C-S (2003). Establishment and application of a fluorescent polymerase chain reaction−restriction fragment length polymorphism (PCR-RFLP) method for identifying porcine, caprine, and bovine meats. J. Agric. Food Chem..

[41] Wallace DC, Chalkia D (2013). Mitochondrial DNA genetics and the heteroplasmy conundrum in evolution and disease. Cold Spring Harb. Perspect. Biol..

[42] Kek SP, Chin NL, Tan SW, Yusof YA, Chua LS (2017). Molecular identification of honey entomological origin based on bee mitochondrial 16S rRNA and COI gene sequences. Food Control.

[43] Wilwet L, Jeyasekaran G, Shakila RJ, Sivaraman B, Padmavathy P (2018). A single enzyme PCR-RFLP protocol targeting 16S rRNA/tRNA(val) region to authenticate four commercially important shrimp species in India. Food Chem..

[44] Wang Q (2022). Research progress on mutton origin tracing and authenticity. Food Chem..

[45] Zeng L (2018). Identification of sea cucumber species in processed food products by PCR-RFLP method. Food Control.

[46] Sforza S, Corradini R, Tedeschi T, Marchelli R (2011). Food analysis and food authentication by peptide nucleic acid (PNA)-based technologies. Chem. Soc. Rev..

[47] Anjali KM (2019). Identification of six grouper species under the genus *Epinephelus* (Bloch, 1793) from Indian waters using PCR-RFLP of cytochrome c oxidase I (COI) gene fragment. Food Control.

