# Supplementary material for: An innovative molecular approach towards the cost-effective entomological authentication of honey
Source: NPJ Sci Food. 2024 May 2;8:24. doi: 10.1038/s41538-024-00268-9 (PMC11063038; doi:10.1038/s41538-024-00268-9)
Supplement: Supplementary file 1 — Supplementary Information [file 41538_2024_268_MOESM1_ESM.pdf]

Supplementary Information

Supplementary Table 1. Sample information of *Apis mellifera* honeys in China (n=30).

Botanical source	Date	Sampling spot	Number	Notes
Sapindus saponaria L.	June, 2014	Jinhua City, Zhejiang Province	3	
Polyflora	April, 2015	Xi'an City, Shaanxi Province	1	
Citrus maxima (Burm) Merr.	April, 2015	Quzhou City, Zhejiang Province	1	
Ilex chinensis Sims	June, 2015	Jinhua City, Zhejiang Province	1	
Tilia amurensis Rupr.	July, 2016	Baishan City, Jilin Province	2	
Ziziphus jujuba Mill.	January, 2017	Hangzhou City, Zhejiang Province	2	
Glochidion puberum (L.) Hutch.	July, 2017	Ningbo City, Zhejiang Province	1	
Litchi chinensis Sonn.	April, 2018	Shenzhen City, Guangdong Province	2	
Brassica napus L.	April, 2018	Xinghua City, Jiangsu Province	1	
Foeniculum vulgare Mill.	July, 2020	Wuwei City, Gansu Province	3	
Brassica napus L.	March, 2020	Quzhou City, Zhejiang Province	1	
Ziziphus jujuba Mill.	May, 2020	Yan'an City, Shaanxi Province	2	
Scrophularia ningpoensis Hemsl.	July, 2020	Bozhou City, Anhui Province	3	
Ziziphus jujuba Mill.	August, 2021	Hangzhou City, Zhejiang Province	1	
Polyflora	November, 2021	Shaoxing City, Zhejiang Province	1	
Carthamus tinctorius L.	May, 2022	Hangzhou City, Zhejiang Province	3	Commercial honey
Robinia pseudoacacia L.	May, 2022	Yan'an City, Shaanxi Province	1	
Brassica napus L.	April, 2022	Jinhua City, Zhejiang Province	2	

Supplementary Table 2. Sample information of *Apis cerana* honeys in China (n=30).

Botanical source	Date	Sampling spot	Number
Polyflora	November, 2017	Shennongjia Forestry District, Hubei Province	2

Litchi chinensis Sonn.	April, 2018	Shenzhen City, Guangdong Province	2
Brassica napus L.	April, 2018	Xinghua City, Jiangsu Province	1
Brassica napus L.	March, 2020	Quzhou City, Zhejiang Province	2
Polyflora	May, 2020	Hangzhou City, Zhejiang Province	2
Ziziphus jujuba Mill.	June, 2020	Yan'an City, Shaanxi Province	3
Rhus chinensis Mill.	June, 2020	Yan'an City, Shaanxi Province	2
Polyflora	November, 2020	Shennongjia Forestry District, Hubei Province	4
Eurya japonica Thunberg	March, 2021	Shangrao City, Jiangxi Province	1
Polyflora	April, 2021	Wenzhou City, Zhejiang Province	1
Triadica cochinchinensis Loureiro	May, 2021	Shangrao City, Jiangxi Province	1
Leucosceptrum canum Smith	May, 2021	Dehong Dai and Jingpo Autonomous Prefecture, Yunnan Province	2
Polyflora	November, 2021	Enshi City, Hubei Province	2
Eriobotrya japonica (Thunb.) Lindl.	December, 2021	Hangzhou City, Zhejiang Province	3
Polyflora	March, 2022	Hangzhou City, Zhejiang Province	1
Polyflora	May, 2022	Chongzuo Municipality, GuangXi Zhuang Autonomous Region	1
Polyflora	June, 2022	Aba (Ngawa) Tibetan and Qiang Autonomous Prefecture, Sichuan Province	1

Supplementary Table 3. Sample information of *Apis laboriosa* honeys in China (n=30).

Botanical source	Date	Sampling spot	Number
Polyflora	April, 2020	Honghe Hani and Yi Autonomous Prefecture, Yunnan Province	4
Polyflora	May, 2020	Puer City, Yunnan Province	1
Polyflora	May, 2020	Lincang City, Yunnan Province	3
Polyflora	May, 2020	Tibet autonomous region	1
Polyflora	April, 2021	Dai Autonomous Prefecture of Xishuangbanna, Yunnan Province	1
Polyflora	May, 2021	Dehong Dai and Jingpo Autonomous Prefecture, Yunnan Province	12
Polyflora	April, 2022	Dehong Dai and Jingpo Autonomous Prefecture, Yunnan Province	5
Polyflora	April, 2022	Dai Autonomous Prefecture of Xishuangbanna, Yunnan Province	3

Supplementary Table 4. Sample information of *Apis dosata* honeys in China (n=30).

Botanical source	Date	Sampling spot	Number
Polyflora	May, 2020	Hainan Province	2
Polyflora	June, 2020	Dai Autonomous Prefecture of Xishuangbanna, Yunnan Province	7
Polyflora	June, 2020	Guangxi Province	1
Polyflora	April, 2021	Dai Autonomous Prefecture of Xishuangbanna, Yunnan Province	4
Polyflora	June, 2021	Dai Autonomous Prefecture of Xishuangbanna, Yunnan Province	4
Polyflora	April, 2022	Dai Autonomous Prefecture of Xishuangbanna, Yunnan Province	10
Polyflora	May, 2022	Chongzuo City, Guangxi Province	1
Polyflora	May, 2022	Baise City, Guangxi Province	1

Supplementary Table 5. Sample information of *Apis florea* honeys in China (n=26).

Botanical source	Date	Sampling spot	Number
Polyflora	June, 2020	Dai Autonomous Prefecture of Xishuangbanna, Yunnan Province	2
Polyflora	July, 2020	Honghe Hani and Yi Autonomous Prefecture, Yunnan Province	2
Polyflora	April, 2021	Dai Autonomous Prefecture of Xishuangbanna, Yunnan Province	5
Polyflora	June, 2021	Dai Autonomous Prefecture of Xishuangbanna, Yunnan Province	4
Polyflora	April, 2022	Chongzuo City, Guangxi Province	2
Polyflora	May, 2022	Chongzuo City, Guangxi Province	3
Polyflora	May, 2022	Yuxi City, Yunnan Province	2
Polyflora	June, 2022	Honghe Hani and Yi Autonomous Prefecture, Yunnan Province	2
Polyflora	September, 2022	Yuxi City, Yunnan Province	1
Polyflora	September, 2022	Honghe Hani and Yi Autonomous Prefecture, Yunnan Province	3

Supplementary Table 6. Sample information of *Apis andreniformis* honeys in China (n=2).

Botanical source	Date	Sampling spot	Number
Polyflora	September, 2021	Dai Autonomous Prefecture of Xishuangbanna, Yunnan Province	2
